# The Ontology of Vaccine Adverse Events (OVAE) and its usage in representing and analyzing adverse events associated with US-licensed human vaccines

**DOI:** 10.1186/2041-1480-4-40

**Published:** 2013-11-26

**Authors:** Erica Marcos, Bin Zhao, Yongqun He

**Affiliations:** 1 College of Literature, Science, and the Arts, University of Michigan, Ann Arbor, MI 48109, USA; 2 School of Information, University of Michigan, Ann Arbor, MI 48109, USA; 3 Unit for Laboratory Animal Medicine, University of Michigan Medical School, Ann Arbor, MI 48109, USA; 4 Department of Microbiology and Immunology, University of Michigan Medical School, Ann Arbor, MI 48109, USA; 5 Center for Computational Medicine and Bioinformatics, University of Michigan Medical School, Ann Arbor, MI 48109, USA

## Abstract

**Background:**

Licensed human vaccines can induce various adverse events (AE) in vaccinated patients. Due to the involvement of the whole immune system and complex immunological reactions after vaccination, it is difficult to identify the relations among vaccines, adverse events, and human populations in different age groups. Many known vaccine adverse events (VAEs) have been recorded in the package inserts of US-licensed commercial vaccine products. To better represent and analyze VAEs, we developed the Ontology of Vaccine Adverse Events (OVAE) as an extension of the Ontology of Adverse Events (OAE) and the Vaccine Ontology (VO).

**Results:**

Like OAE and VO, OVAE is aligned with the Basic Formal Ontology (BFO). The commercial vaccines and adverse events in OVAE are imported from VO and OAE, respectively. A new population term ‘human vaccinee population’ is generated and used to define VAE occurrence. An OVAE design pattern is developed to link vaccine, adverse event, vaccinee population, age range, and VAE occurrence. OVAE has been used to represent and classify the adverse events recorded in package insert documents of commercial vaccines licensed by the USA Food and Drug Administration (FDA). OVAE currently includes over 1,300 terms, including 87 distinct types of VAEs associated with 63 human vaccines licensed in the USA. For each vaccine, occurrence rates for every VAE in different age groups have been logically represented in OVAE. SPARQL scripts were developed to query and analyze the OVAE knowledge base data. To demonstrate the usage of OVAE, the top 10 vaccines accompanying with the highest numbers of VAEs and the top 10 VAEs most frequently observed among vaccines were identified and analyzed. Asserted and inferred ontology hierarchies classify VAEs in different levels of AE groups. Different VAE occurrences in different age groups were also analyzed.

**Conclusions:**

The ontology-based data representation and integration using the FDA-approved information from the vaccine package insert documents enables the identification of adverse events from vaccination in relation to predefined parts of the population (age groups) and certain groups of vaccines. The resulting ontology-based VAE knowledge base classifies vaccine-specific VAEs and supports better VAE understanding and future rational AE prevention and treatment.

## Background

Many licensed vaccines exist to protect against a variety of diseases and infections. They are extremely useful in decreasing infection prevalence in human populations. Due to the public health benefits of vaccines, their coverage has been increasing in recent years. However, each vaccine often induces different types of adverse events (AEs). As vaccine usage increases, the risk of adverse events proportionally increases [1]. There is a need to predict probabilities of different adverse events arising in different individuals, which can potentially lead to a decline in the risk of developing an adverse event. Many known vaccine adverse events (VAEs) at the population level have been recorded in the package inserts of commercial vaccine products. The VAE information in the package inserts may be used for systematic VAE analysis and comparison, providing a fundamental basis for further individual level VAE evaluation and prediction.

Two existing ontologies are closely related to the VAE studies. The Ontology of Adverse Events (OAE) is a community-based biomedical ontology in the area of adverse events [2,3]. OAE defines an ‘adverse event’ as a pathological bodily process that occurs after a medical intervention (*e.g.*, vaccination, drug administration). The OAE ‘adverse event’ is a subclass of the ontology term ‘pathological bodily process’ defined in the Ontology of General Medicine Science (OGMS) (http://code.google.com/p/ogms/). To be consistent with most practical uses of the term, OAE does not assume a causal relation between an ‘adverse event’ and a medical intervention. OAE has defined over 2,000 types of adverse events that are commonly found in different medical interventions. The community-based Vaccine Ontology (VO) represents various vaccines, vaccine components, and vaccinations [4,5]. Both OAE and VO are OBO Foundry library ontologies and are developed by following the OBO Foundry principles [6].

OAE has been shown to significantly increase the power of analyzing often noisy case report data from the Vaccine Adverse Event Reporting System (VAERS) [3]. In this study, the adverse events associated with killed attenuated and live attenuated influenza vaccines were separately extracted from VAERS, statistically analyzed, and compared with each other. The AEs annotated and stored in VAERS were assigned to the Medical Dictionary for Regulatory Activities (MedDRA) codes [7]. Compared to MedDRA, OAE was found to be better to classify the groups of AEs associated with different types of influenza vaccines, and biologically significant findings were generated [3]. Due to the lack of randomized, well-controlled studies, it is often difficult to justify the causality between an adverse event reported and a vaccine administration using the VAERS or other clinical case report data. However, the results cited from the package insert documents of FDA licensed vaccines were typically generated from randomized, well-controlled clinical trials. Compared to the noisy data from clinical VAE case reports, the adverse events recorded in the official package inserts are known adverse events specific for individual vaccines. To our knowledge, there has been no published paper in the ontological domain to analyze commonly known VAEs recorded in the FDA package insert documents.

To better represent various VAEs and support vaccine safety study, we developed the Ontology of Vaccine Adverse Events (OVAE) as an extension of the biomedical ontologies OAE and VO. In this paper, we introduce the basic framework of the OVAE and how OVAE is used to represent and analyze all adverse events reported in the product package inserts of 63 FDA approved commercial vaccines currently used in the USA market.

## Results

### OVAE system design and statistics

The goal of current OVAE development is to generate an ontology-based VAE knowledge base that represents known adverse events (AEs) associated with licensed vaccines. Such a knowledge base incorporates the OAE terms of AEs together with the vaccine information defined in the VO. As the primary developer of the OAE and VO, we argue that OAE is not appropriate or responsible for representing various AEs specific for any particular medical intervention including vaccination due to the following reasons. First, OAE emphasizes the representation of various AEs general for most medical interventions, and related topics (*e.g.*, methods for analysis of the causal relation between AEs and medical interventions, and factors affecting the causality analysis). Currently OAE is already large and contains nearly 3,000 terms. It is expected that many more AE terms will be added to OAE. Therefore, it is ideal to make OAE focused and as concise as possible. Secondly, AE researchers related to specific medical intervention domains may have more domain-specific demands and requests. For example, VAE researchers would like to link AEs to different vaccines. The drug researchers may prefer to associate AEs with specific drugs. The vaccine (or drug) researchers may not be interested in drug (or vaccine) specific AEs. As a relatively independent domain, VAEs have been focuses of many vaccine researchers and groups. Independent from drug AEs, clinical VAEs are reported to vaccine-specific VAERS system in the USA [8]. Meanwhile, the Vaccine Ontology (VO) is not suitable for representing complex VAE data. VO has been focused on classification of various vaccines, including licensed vaccines, vaccines in clinical trials, and vaccines only verified in laboratory animal models. VO also represents various types of vaccine components (*e.g.*, vaccine antigens, adjuvants, and vectors), vaccine attributes (*e.g.*, vaccine organism viability and virulence), vaccination methods, and other concise and closely related vaccine information. The inclusion of complex and large VAE information to VO would make VO imbalance and not specific enough. Due to these reasons, we generated the VAE-specific OVAE, which is an extension of OAE and VO. OVAE specifies AEs associated with various vaccines, for example, influenza vaccine Afluria-associated pain adverse event. The logical definition of such a VAE requires both the pain AE term from OAE and the Afluria vaccine term from VO. Such a term cannot be captured without the OVAE. The OVAE integration of OAE and VO is also required to link such a term to related features about the AE and vaccine, for example, the parent term of pain AE and the patient age requirement for the vaccine administration. Since both OAE and VO use the Basic Formal Ontology (BFO) (http://www.ifomis.org/bfo) as the top level class, the alignments between OVAE, OAE, and VO are easy and straightforward.

As an extension of OAE and VO, OVAE targets for not only importing related terms from these two ontologies but also including many OVAE-specific terms. The primary data source for generating vaccine-specific AE ontology terms in current OVAE is the official vaccine package inserts available in the USA FDA website [9]. Each official vaccine package insert document provided by the USA FDA includes a section called “Adverse Reactions”. The results provided in the section were obtained from carefully designed clinical trials with randomized controls and worldwide post-marketing experience. Therefore, the VAE information provides basic known VAEs that are likely to occur after an administration of a specific vaccine in a human vaccinee. Based on the officially documented information, OVAE includes many OVAE-specific terms, for example, ‘Afluria-associated pain AE’ to define a pain AE specific for Afluria-vaccinated patients. As shown in detail later in the paper, the generation of these new terms allows the inclusion of more detailed information about these VAEs, for example, the VAE occurrences in human vaccinee populations in different age groups.

Table 1 lists the OVAE statistics as of July 1, 2013. OVAE used the most recent BFO 2.0 Graz version (http://purl.obolibrary.org/obo/bfo.owl) as the top level ontology. Since BFO 2.0 is not yet finalized, some relation terms (*e.g.*, ‘part of’ or BFO_0000050) are still used in OVAE but do not necessarily comply with the most recent BFO 2.0. During the process of importing many AEs or vaccine-related terms from OAE and VO to OVAE, many terms from other existing ontologies, including OGMS, Ontology for Biomedical Investigation (OBI) [10], Phenotypic Quality Ontology (PATO) [11], and Information Artifact Ontology (IAO) (http://code.google.com/p/information-artifact-ontology/), have also been imported to OVAE (Table 1). To maintain the ontology asserted and inferred hierarchies and support intact reasoning capability, the OntoFox software was used for external term importing [12]. In summary, OVAE contains 1,327 terms, including 626 OVAE-specific terms (with “OVAE_” prefix). In addition, OVAE includes many ontology terms from external ontologies, for example, all 128 terms from the BFO version 2.0, 197 VO terms, 120 OAE terms, 16 OBI terms, 6 IAO terms, and 2 OGMS terms (Table 1). By referencing the vaccine package insert data, OVAE represents 87 distinct AEs associated with 63 licensed human vaccines.

**Table 1:** Summary of ontology terms in OVAE

**Ontology names**	**Classes**	**Object properties**	**Data properties**	**Total**
OVAE	626	1	1	628
BFO	40	88	0	128
CHEBI	7	0	0	7
OBI	8	8	0	16
PATO	7	0	0	7
IAO	6	0	0	6
NCBOTaxon	81	0	0	81
OAE	119	1	0	120
OGMS	2	0	0	2
RO	0	15	0	15
UBERON	105	15	0	220
VO	188	6	3	197
**Total:**	1189	124	4	1,327

### OVAE design pattern of representing VAE

**Figure 1:**
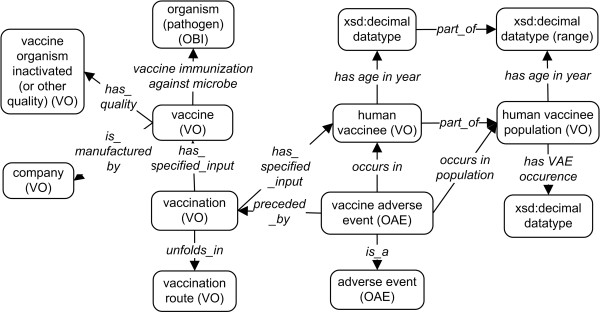
OVAE design pattern of a human vaccine adverse event.

The general design pattern of representing a VAE in OVAE is shown in Figure 1. Specifically, a licensed vaccine, manufactured by a company and having specific quality (*e.g.*, using inactivated vaccine organism), is targeted to immunize a human vaccinee against infection of a microbial pathogen. A particular vaccination route (*e.g.*, intramuscular route) is specified. A specific VAE (*e.g.*, Afluria-associated injection-site pain adverse event) occurs in a human vaccinee and after (*preceded_by*) a vaccination. The human vaccinee, having a specific age (defined via a datatype) at the time of vaccination, is part of the population of human vaccinees using this vaccine. The VAE occurrence is defined as a frequency of an adverse event associated with the administration of a vaccine in a vaccinee population. The new object property term ‘has VAE occurrence’ is defined in OVAE to specify a VAE occurrence (xsd:decimal datatype) in a human vaccinee population that has been individually vaccinated with a specific vaccine during a specific time period. To simplify the representation of axioms linking vaccine adverse event and human vaccinee population, OVAE generates a shortcut relation ‘occurs in population’ (Figure 1).

The vaccine attributes and vaccination details are imported from VO. Their inclusion in the design pattern is due to their possible contribution to the VAE determination. For example, a live attenuated vaccine and a killed inactivated vaccine may in general induce different types or levels of VAEs, which can be analyzed by statistical analysis [3].

One novelty in the design pattern is the generation and application of the population term ‘human vaccinee population’ to define a VAE occurrence. In previous versions of OAE and VO, only ‘vaccinee’ and ‘human vaccinee’ (*i.e.*, a human being administered with a vaccine) exist. However, it is incorrect to say that a specific human vaccinee has a VAE occurrence of some percentage (*e.g.*, 10%). An occurrence is defined only for a population. The generation of the term ‘human vaccinee population’ solves the ontology modeling issue. Any particular human vaccinee is part of a human vaccinee population.

**Figure 2:**
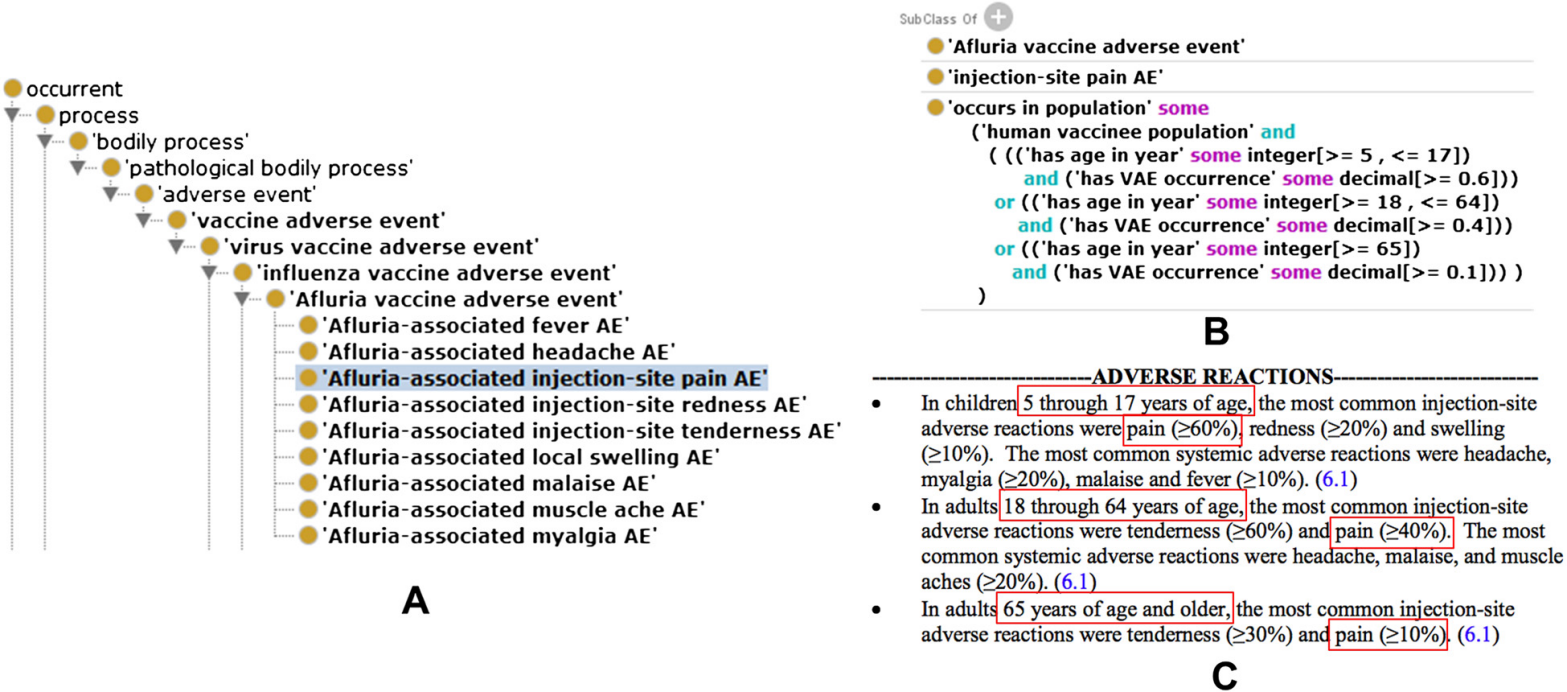
**OVAE representing Afluria VAEs reported in FDA vaccine package insert. (A)** The hierarchical structure of Afluria VAEs represented in OVAE. **(B)** OVAE axiom representation of ‘Afluria-associated injection-site pain AE’ based on three age groups. **(C)** Afluria adverse reactions recorded in the FDA package insert document. Other VAEs shown in the FDA package inserts are also represented in OVAE. The subfigures **(A)** and **(B)** were screenshots of OVAE using the Protégé OWL editor. The text from **(C)** comes from the FDA package insert document of the Afluria vaccine.

There are two different approaches for representing the relation between a human vaccinee (or human vaccinee population) and an age (or age range). One approach is to link a vaccinee to a quality named ‘age’ , and then link the ‘age’ to a datatype using the OBI relation term ‘quality measured as’. Another approach for representing the relation is to generate a shortcut relation ‘has age’ (or specifically ‘has age in year’). To make the representation simpler and reasoning efficient, we have taken the second choice. The use of the relation ‘has age’ will need to specify the data value as well as the unit of the data (*e.g.*, year). The use of the shortcut relation ‘has age in year’ is much simpler, requiring only the data value. An example is provided below (Figure 2).

### Generation of OVAE covering FDA package insert AE information

Based on the design pattern described above, the OVAE was generated to cover the AE information extracted from the FDA package insert documents [9]. The FDA website includes supporting materials for most human vaccines licensed in the USA [9]. The detailed methods of how to manually annotate the VAE information and represent the knowledge in OVAE are described in the Methods section.

An example of OVAE representation of VAE is shown in Figure 2. Briefly, Afluria has been associated with nine different types of AEs, including injection-site pain AE that has been defined in OAE (Figure 2A and 2B). For each AE, it is likely that different VAE occurrences are reported based on the age groups. OVAE uses two datatype property terms (‘has age in year’ and ‘has VAE occurrence’) to link vaccinee population groups and VAEs associated with particular VAE occurrences (Figure 2B). The “OR” clause is used to include vaccinee populations with different age ranges. The information matches to the FDA package insert information (Figure 2C). The FDA package insert citation was also used as a definition source (annotation property).

### SPARQL query of OVAE data

The SPARQL Protocol and RDF Query Language (SPARQL) is a query language for querying and manipulating data stored in a RDF tripe store. SPARQL is a standard recommended by the World Wide Web Consortium (W3C), and is recognized as a key technology of the Semantic Web. SPARQL 1.1 has been the official version since March, 2013 [13]. SPARQL queries allow for triple patterns, conjunctions, disjunctions, and optional patterns.

**Figure 3:**
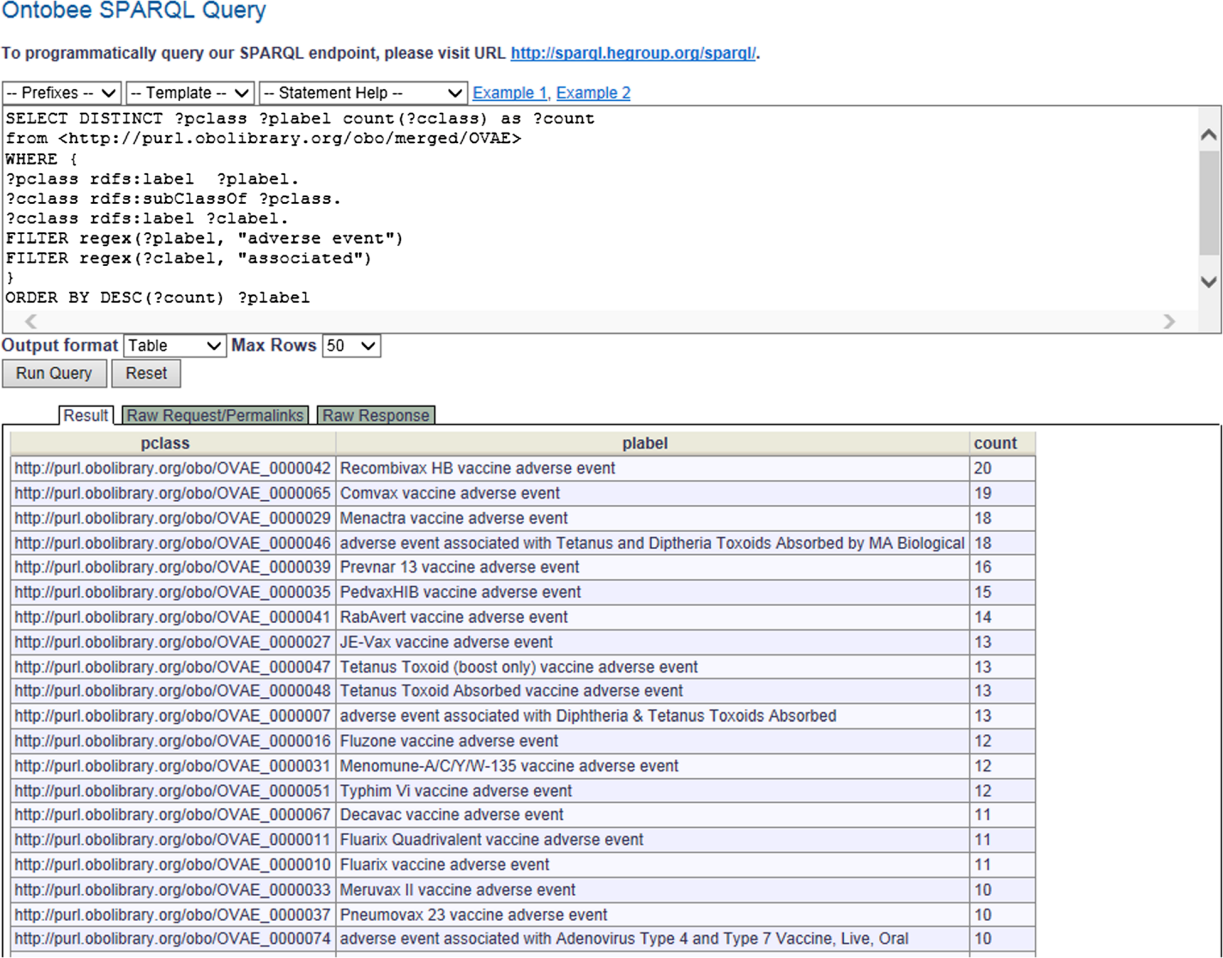
**Example SPARQL for OVAE query.** This example queries the numbers of specific adverse events associated with individual vaccines. The SPARQL script is shown at the top. Below the script is the results obtained after execution of the SPARQL query. See the text in the manuscript for detailed explanation.

Figure 3 demonstrates an example of how to use SPARQL to count the number of specific adverse events for each vaccine. Figure 3A is a SPARQL script for querying OVAE in a RDF triple store. In this SPARQL query, the source of the OVAE ontology is specified following the “FROM” keyword. In this script, the variables “?pclass” and “?cclass” are two classes with their labels (rdfs:label) “?plabel” and “?clabel”, respectively. The child class “?cclass” is a subclass (rdfs:subClassOf) of the parent class “?pclass”. A regular expression (regex) filter function requires that the string “?plabel” include the words “adverse events”, for example, “Recombivax HB vaccine adverse event”. Another regex filter function specifies the inclusion of the word “associated” in the subclass label “?clable”, for example, “Recombivax HB-associated fever AE”. These two regex functions are designed based on the naming convention defined in OVAE. Specifically, a bottom-level vaccine-specific adverse event term label always uses the words “associated” and “AE” (instead of “adverse event”), and its parent vaccine-specific term label always contains the words “adverse event” (instead of the abbreviation version “AE”). To display the results, the SELECT function in the script specifies “?pclass”, “?plabel”, and the total count of “?cclass” in a decreasing order (“DESC”) based on the count. The top eight query results are shown in Figure 3B.

In this study, different SPARQL scripts were generated to address different questions as exemplified below.

### OVAE VAE data analysis results

After all VAEs found in FDA licensed vaccines are represented in OVAE, the quality of the ontology was manually annotated, and an ontology reasoner [14] was used to ensure no logical errors occurring in the ontology formation. To address scientific questions associated with different vaccine AEs, the OVAE ontology was queried using SPARQL. The Protege-OWL editor also provides user-friendly function to directly visualize OVAE results. Below we provide examples to illustrate how the analysis of the OVAE knowledge base can be used to answer different VAE questions.

First, those vaccines that are associated with the largest number of VAEs were analyzed (Table 2). It is interesting that many of these vaccines protect against meningitis, which can be caused by different pathogens including *Haemophilus influenza* type b (Comvax and PedvaxHIB), *Neisseria meningitides* (Menactra), and *Streptococcus pneumonia* (Prevnar 13). The list also includes three tetanus vaccines and two Hepatitis B vaccines (Table 2). The relation between these common diseases/pathogens and the high variety of VAEs reported is unclear and deserves further investigations. It is noted that the information does not dictate the severity of AEs associated with each vaccine, but instead indicates that those FDA-licensed vaccines display the most variation in their reported AEs.

**Table 2:** Top 10 vaccines with the largest variety of VAEs reported

**Vaccine (disease or pathogen)**	**VO_ID**	**Total # VAE**
Recombivax HB (Hepatitis B)	VO_0010737	20
Comvax (Hib meningitis, Hepatitis B)	VO_0000028	19
Menactra (*Neisseria meningitidis*)	VO_0000071	18
Tetanus and Diphtheria Toxoids	VO_0000111	18
Absorbed by MA Biological		
(Tetanus, Diphtheria)		
Prevnar 13 (*Streptococcus pneumonia*)	VO_0000090	16
PedvaxHIB (*H. influenzae* type b)	VO_0000083	15
RabAvert (Rabies)	VO_0000094	14
JE-Vax (Japanese Encephalitis)	VO_0000066	13
Tetanus Toxoid (boost only) (Tetanus)	VO_0000984	13
Tetanus Toxoid Absorbed (Tetanus)	VO_0000048	13

**Note:** The disease or pathogen name specified next to a vaccine name indicates the disease or pathogen infection against which the vaccine is used. The three bacteria listed (shown by italicized words) can all induce meningitis. The vaccines are sorted based on the VAEs recorded in their package insert documents.

Secondly, we evaluated the top VAEs that have been reported most frequently among all vaccines licensed in the USA and represented by OVAE (Table 3). Most of the top 10 frequently observed VAEs are expected, such as injection-site pain and redness, fever, and local swelling. The headache and myalgia (*i.e.*, muscle pain) are two subtypes of pain. The pain AE, malaise (*i.e.* uneasiness and discomfort) AE, and fatigue AE are all subtypes of behavior and neurological AEs. The frequent occurrence of behavior and neurological AE is likely associated with the common intramuscular route used for vaccine administration. Specific microbial antigen contents may also induce frequently observed VAEs (*e.g.*, fever). It is noted that the information does not dictate which VAEs are the most severe, but indicates which VAEs are commonly observed in currently licensed vaccines in the USA.

**Table 3:** Top 10 most frequently reported VAEs

**AE name**	**OAE_ID**	**Total # vaccines**	**%**
Injection-site pain AE	OAE_0000369	43	68.3
Headache AE	OAE_0000377	39	61.9
Fever AE	OAE_0000361	34	54.0
Local swelling AE	OAE_0001139	30	47.6
Injection-site redness AE	OAE_0001546	25	40.7
Irritability AE	OAE_0001105	23	36.5
Malaise AE	OAE_0000390	21	33.3
Injection-site erythema AE	OAE_0000644	20	31.7
Myalgia AE	OAE_0000375	19	30.2
Fatigue AE	OAE_0000034	18	28.6

**Figure 4:**
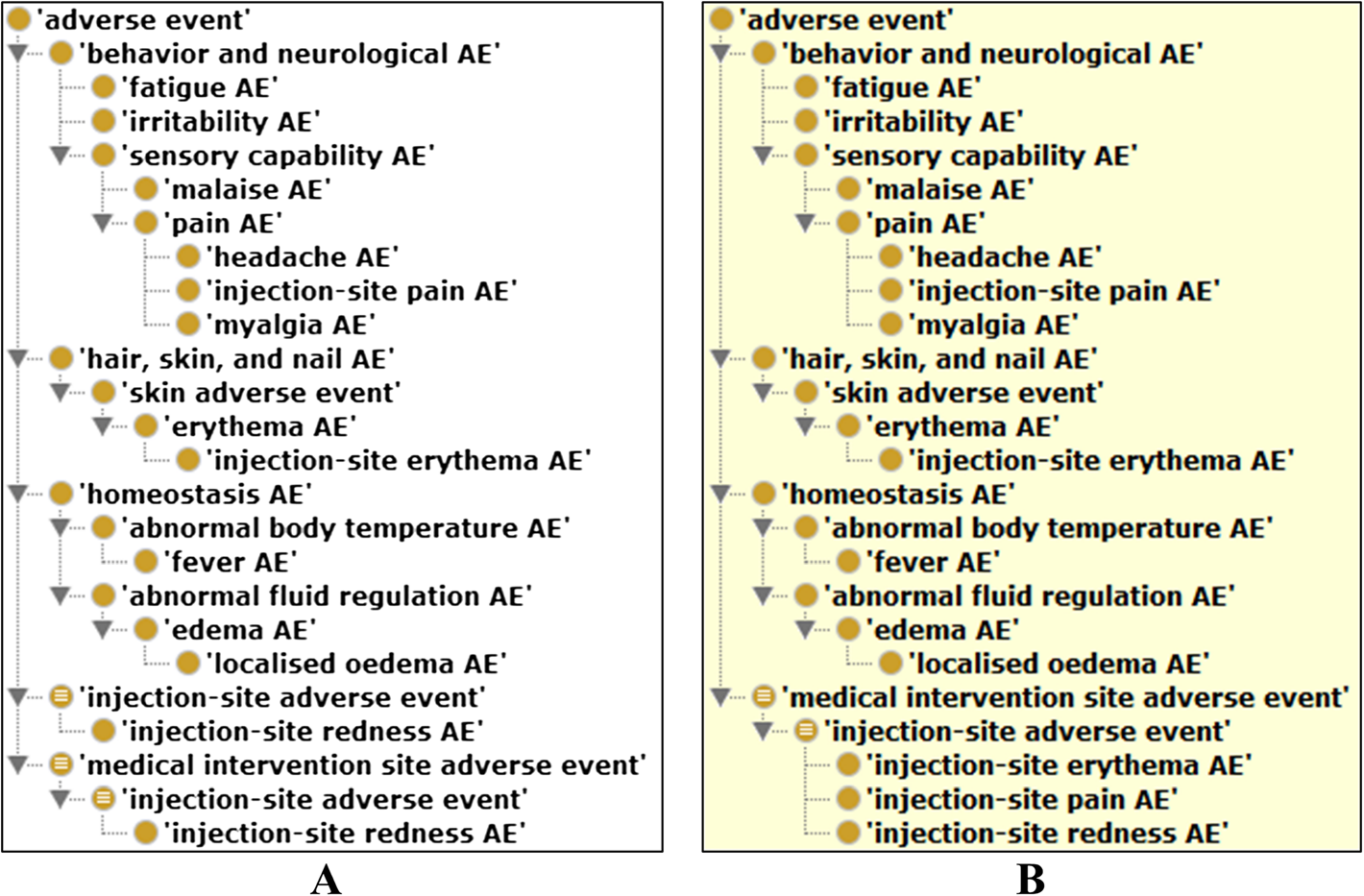
**Classification of top 10 AEs associated with licensed human vaccines in the US.** These OAE terms have been imported to OVAE using OntoFox and visualized using Protégé OWL editor. **(A)** Asserted hierarchy in OAE; **(B)** Inferred hierarchy after reasoning.

To better understand the top VAEs associated with licensed human vaccines, the hierarchical structure of the top 10 VAEs (Table 3) was extracted using the tool OntoFox and visualized using Protégé ontology editor (Figure 4). The hierarchical visualization indicates that most of the top ranked VAEs belong to the behavior and neurological AE branch. It is also noted that after reasoning, two adverse events (*e.g.*, injection-site pain AE) were inferred to be subclasses of ‘injection-site adverse event’ (Figure 4B). Since OAE does not allow multiple inheritance, injection-site pain AE cannot be asserted under both ‘pain AE’ and ‘injection-site adverse event’. In OAE, injection-site pain AE is asserted under ‘pain AE’ which occurs in an injection site. A reasoner will be able to infer it as a subclass of ‘injection-site adverse event’ as well (Figure 4B). The ontology reasoning provides additional power in VAE classification.

Furthermore, we compared the VAEs and VAE occurrences under different age groups. As shown in Figure 2, the OVAE clearly represents the associations between VAEs, the VAE occurrence rates, and different ages (in years) of human vaccinee population. Our analysis can further identify which age category has a higher probability of experiencing any specific adverse events. For example, we found that *Salmonella typhi* vaccine Typhim Vi is associated with injection-site tenderness adverse events with the highest rate of 97.5% at the age group of 18–40 years old. Based on the classification of “child”, “adult”, and “child-adult” described in the Methods section in the paper, there are 240, 160, and 177 vaccine-specific AEs in the age categories “child”, “adult”, and “child-adult”, respectively. It is also found that in general the VAE occurrences shown in the children are typically higher than those in adults. This suggests that individuals under 18 years may be more likely to experience an adverse reaction after vaccination.

The above examples illustrate the advantages of OVAE in VAE data integration and analyses. The usage of OVAE provides an efficient approach to answer different VAE questions, which would be very difficult to address without such an ontology.

## Discussion

The development of OVAE is aimed to align and reuse existing ontologies OAE and VO, and systematically represent and analyze vaccine-specific adverse events (VAEs). As demonstrated in this report, such a strategy has many advantages. First, as shown in Figure 2, the ontological classification is easy for humans to interpret and analyze. A human can browse the hierarchical tree to quickly understand which VAEs are typically associated with a licensed vaccine. Secondly, the ontology OWL representation is also interpretable by computers and software programs. New programs can be developed to parse and analyze the information. Thirdly, the approach of aligning OVAE with existing ontologies allows efficient integration of data presented in other ontologies (*e.g.*, VO). Fourthly, the usage of OVAE and other related ontologies makes it possible to analyze VAEs with various tools such as VO-based literature mining [15]. Eventually, an ontology-based linked VAE data system can be generated.

Furthermore, it is possible to apply the OVAE framework to analyze clinical VAE data such as those case reports stored in VAERS [8]. For example, by comparing the reported vaccine-specific VAE cases in VAERS with the VAE occurrences reported in the package inserts and OVAE, it is easy to differentiate known VAEs and possibly new VAEs associated with the vaccine. Many differences exist in terms of the data shown in the package inserts and in VAERS database. While the data in the package inserts were typically obtained from well controlled clinical trials, clinical VAE case reports stored in VAERS came from random reports from physicians, patients, patients’ parents, or other sources. The VAERS database does not indicate the total number of vaccinated human vaccinees in any given period, making it impossible to calculate exact VAE occurrences. However, an ontological approach in combination with a statistical analysis is still useful in VAERS data analysis as previously demonstrated [3]. Currently the AE data stored in VAERS are annotated using the Medical Dictionary for Regulatory Activities (MedDRA), a coding vocabulary nomenclature commonly used for clinical adverse event recording and normalization [16]. However, many disadvantages of MedDRA, including the lack of term definitions and a well-defined hierarchical and logical structure, prevent its effective usage in VAE term classification. Our previous study showed that a mapping between MedDRA and OAE terms followed by the application of OAE hierarchy provided a feasible solution for valid classification of VAEs detected through statistical analyses of VAERS data [3]. MedDRA does not have rich axiomatization as shown in OAE and OVAE. The richer and verified ontological axiomatization will facilitate VAE data analysis. As an extension of both OAE and VO, OVAE represents various VAEs associated with different licensed vaccines. One future research direction will be to identify novel ways to better analyze VAE clinical data using OVAE. Indeed, one effective way is to develop an OVAE-based "Linked Data" (LD; http://www.w3.org/standards/semanticweb/data) system specifically for representing and sharing various VAE clinical and research "instance" data obtained from VAERS and other resources. Advanced reasoning methods can then be developed to analyze the large but well-organized data in the linked data system. Such an strategy is being designed and implemented in our group.

While many AEs are common, different vaccines are associated with different AEs with various molecular mechanisms. The classification of different vaccine-specific AEs allows us to examine the similarities and difficulties of molecular interactions and pathways underlying different types of VAEs. Various Omics and informatics tools can also be applied. Therefore, the ontology representation of vaccine-specific AEs is a first step towards refined deep understanding of vaccine adverse events. The better understanding of the vaccine-specific AE patterns and the underlying molecular mechanisms will make it possible to rationally design practical measures to prevent and treat VAEs and thus support public health.

In addition to the VAEs associated with USA licensed vaccines, the OVAE can be used to represent VAEs associated with vaccines licensed in other countries. It is also noted that the method of establishing vaccine-specific OAE extension may likely be applied for developing OAE extensions in other specified domains such as drug-associated adverse events.

## Conclusions

The Ontology of Vaccine Adverse Events (OVAE) ontologically represents and classifies various identified vaccine adverse events (VAEs) associated with human vaccines licensed for use in the USA. Systematical analysis of the OVAE data improves the understanding of vaccine-specific VAEs, making it possible to rationally design VAE prevention and treatment measures and to benefit public health.

## Methods

### OVAE ontology generation

Following VO and OAE, OVAE is also edited with the Web Ontology Language (OWL2) format (http://www.w3.org/TR/owl-guide/). FDA-licensed human vaccines represented in VO were imported to OVAE using the tool OntoFox [12]. Those adverse event terms reported in the package inserts of FDA licensed human vaccines were also imported to the OVAE using OntoFox. New OVAE-specific terms were generated with IDs containing the prefix of “OVAE_” followed by seven auto-incremental digital numbers and edited using the Protégé 4.2 OWL ontology editor (http://protege.stanford.edu/). The Java-based ELK OWL 2 reasoner [14] was used for OVAE ontology reasoning.

### Data source of known VAEs

The official FDA website that provides supporting documents of licensed vaccines was the primary data source [9]. A PDF version of a package insert document is available for almost every vaccine in the data source. The PDF document includes a section called “Adverse Reactions” that contains text descriptions of known vaccine adverse events associated with the vaccinated population.

### Data collection and formatting to ontology

Based on the OVAE framework and the adverse event description in the package inserts, a design pattern was first generated to lay out the relations between different ontology classes, properties, terms and data types (Figure 1). The design pattern was used to form an MS Excel template for collection of individual adverse events for different vaccines. The MS Excel template includes the following categories: vaccine name, vaccine VO ID, VAE location, VAE name in package insert, VAE name in OAE, OAE ID, age category, age years, VAE occurrence, and reference. Data for each category was manually collected from individual vaccine package inserts and then input into the Excel template. The VAE location is listed as either injection-site or systemic. The injection-site location is incorporated as part of the OAE term, while the systemic AEs are set up as default. Age categories included child (typically under 18 years old), adult (above 18 years old), senior (above 65 years old), or child-adult (all ages). Specific ages are concerted to years and presented to comply with the OWL format. Each VAE is referenced by the package insert citation. Following the manual data collection and annotation, the program Ontorat (http://ontorat.hegroup.org) was used to transform the Excel file data to the OVAE ontology format [17].

### VAE data analysis

To identify specific OAE or VO hierarchical structure among a list of terms, OntoFox was first used to extract the input OAE or VO terms and all associated terms required for proper hierarchical assertion and inference. The output OWL files were then visualized using a Protégé OWL editor.

SPARQL scripts were generated to query the OVAE knowledge base from a RDF triple store that contains the OVAE RDF triples. As an ontology in the OBO Foundry ontology library (http://obofoundry.org/), OVAE is automatically deposited in the Hegroup RDF triple store [18]. The Hegroup triple store, the default OBO Foundry library ontology RDF triple store, is used by Ontobee [18] and can be queried through the Ontobee SPARQL query interface (http://www.ontobee.org/sparql/). Our SPARQL scripts were executed using the Ontobee SPARQL query interface.

To identify specific OAE or VO hierarchical structure among a list of terms, OntoFox was first used to extract the input OAE or VO terms and all associated terms required for proper hierarchical assertion and inference. The output OWL files were then visualized using a Protégé OWL editor.

### OVAE project site, ontology dissemination, and licensing

The OVAE project website (http://www.violinet.org/ovae) is located under VIOLIN, a comprehensive vaccine database and analysis system [19]. OVAE has been deposited to the BioPortal project of the National Center of Biomedical Ontology (NCBO) (http://bioportal.bioontology.org/ontologies/3227). OVAE is also deposited in the Ontobee linked data server (http://www.ontobee.org/browser/index.php?o=OVAE) [18]. The OVAE source code is available in a Google Code website: http://code.google.com/p/ovae. The OVAE source is freely available under the Apache License 2.0.

## Abbreviations

AE: Adverse event; FDA: Food and Drug Administration; NCBO: The National Center for Biomedical Ontology; OAE: Ontology of adverse events; OBI: Ontology for Biomedical Investigations; OBO: The Open Biological and Biomedical Ontologies; OGMS: Ontology for General Medical Science; OVAE: Ontology of Vaccine Adverse Events; OWL: Web Ontology Language; PATO: Phenotypic Quality Ontology; PHP: Hypertext preprocessor; RDF: Resource Description Framework; SPARQL: SPARQL Protocol and RDF Query Language; VAE: Vaccine adverse event; VAERS: Vaccine Adverse Event Reporting System; VIOLIN: Vaccine Investigation and Online Information Network; VO: Vaccine ontology.

## Competing interests

The authors declare that they have no competing interests.

## Authors’ contributions

EM: Vaccine adverse event data annotation and organization, data analysis, and manuscript editing. BZ: SPARQL script development, and manuscript editing. YH: Primary OVAE developer, OVAE design pattern generation, data analysis, and drafting of manuscript. All authors read and approved the final manuscript.
